# Computational analyses reveal fundamental properties of the AT structure related to thrombosis

**DOI:** 10.1093/bioadv/vbac098

**Published:** 2022-12-23

**Authors:** Tiago J S Lopes, Ricardo A Rios, Tatiane N Rios, Brenno M Alencar, Marcos V Ferreira, Eriko Morishita

**Affiliations:** Center for Regenerative Medicine, National Center for Child Health and Development Research Institute, Tokyo 157-8535, Japan; Institute of Computing, Federal University of Bahia, Salvador 40170-110, Brazil; Institute of Computing, Federal University of Bahia, Salvador 40170-110, Brazil; Institute of Computing, Federal University of Bahia, Salvador 40170-110, Brazil; Institute of Computing, Federal University of Bahia, Salvador 40170-110, Brazil; Department of Clinical Laboratory Science, Division of Health Sciences, Kanazawa University, Kanazawa, Ishikawa 920-8641, Japan

## Abstract

**Summary:**

Blood coagulation is a vital process for humans and other species. Following an injury to a blood vessel, a cascade of molecular signals is transmitted, inhibiting and activating more than a dozen coagulation factors and resulting in the formation of a fibrin clot that ceases the bleeding. In this process, antithrombin (AT), encoded by the SERPINC1 gene is a key player regulating the clotting activity and ensuring that it stops at the right time. In this sense, mutations to this factor often result in thrombosis—the excessive coagulation that leads to the potentially fatal formation of blood clots that obstruct veins. Although this process is well known, it is still unclear why even single residue substitutions to AT lead to drastically different phenotypes. In this study, to understand the effect of mutations throughout the AT structure, we created a detailed network map of this protein, where each node is an amino acid, and two amino acids are connected if they are in close proximity in the three-dimensional structure. With this simple and intuitive representation and a machine-learning framework trained using genetic information from more than 130 patients, we found that different types of thrombosis have emerging patterns that are readily identifiable. Together, these results demonstrate how clinical features, genetic data and *in silico* analysis are converging to enhance the diagnosis and treatment of coagulation disorders.

**Supplementary information:**

Supplementary data are available at *Bioinformatics Advances* online.

## 1 Introduction

Blood coagulation is a fundamental mechanism to stop bleeding following damage to a blood vessel. Following an injury to the endothelial cell layer surrounding blood vessels, tissue factor pathway inhibitor (TFPI) is produced and triggers a cascade of molecular signals that involve activation, complex formation and inhibition of more than a dozen proteins and results in the formation of a stable fibrin clot at the site of injury (Lichtman *et al.*, 2021). In this elegant and complex system, it is unsurprising that perturbations to any of its components lead to the disruption of this vital function; for instance, inherited or spontaneous mutations to coagulation factors 8 or 9 lead to hemophilia, a coagulation disorder that causes patients to have uncontrolled bleeding episodes. On the other hand, patients carrying mutations on the SERPINC1 gene are prone to develop thrombosis, a condition characterized by the excessive—and often fatal—formation of blood clots that obstruct veins and arteries (Lichtman *et al.*, 2021).

In humans, the SERPINC1 gene is located at chromosome 1 (1q 23–25), with 13.5 kb distributed in six introns and seven exons (Lichtman *et al.*, 2021) and encodes antithrombin (AT) protein—a master regulator of blood coagulation. The AT protein has 432 amino acids and is a serine protease responsible for inactivating several coagulation factors and acting as a molecular ‘switch’ to ensure that the coagulation process ceases at the right moment. Among its main targets are the activated coagulation factor IX (FIXa), activated coagulation factor X (FXa) and thrombin. AT binds to these targets via a reactive site (RS) at its C-terminus, and this reaction is catalyzed several thousand fold by the presence of heparin, a pentasaccharide that binds to the heparin binding site (HBS) of AT and exerts a strong stabilizing effect on its conformation (reviewed in Huntington, 2011).

These features were revealed by the efforts of several groups that in the last four decades introduced mutations to the SERPINC1 gene (Liu *et al.*, 2014), determined the structure of its protein at different conformations and collected information about the mutations harbored by hundreds of patients with different types of thrombosis (Lane *et al.*, 1994) [Type I deficiencies are defined by decreased protein levels with comparable decreases in protein function. Type II deficiencies have normal or near-normal antigen levels but reduced activity (Kuhle *et al.*, 2001; Smith *et al.*, 2021)]. Interestingly, some groups started using computational techniques to study the effects of mutations in the AT protein using a variety of techniques, including molecular dynamics (Gindele *et al.*, 2021), the prediction of mutation effects (Luxembourg *et al.*, 2011, 2015; Mulder *et al.*, 2017) and explored possible heparin substitutes with higher AT affinities (Navarro-Fernández *et al.*, 2012). Although these studies represent an important step toward the digitalization and potential simulation of the AT protein features *in silico*, their agreement with *in vitro* and clinical data was not satisfactory; their findings were often restricted to a few representative variants of the AT protein, could not be generalized to other known mutations and neither be used to predict the effect of novel mutations.

In this work, we used a known computational technique to represent and study the characteristics of the AT protein. We created a residue interaction network (RIN) of this protein, where the amino acids are the nodes and two nodes are connected if they are in close proximity in the protein’s 3D structure. This type of network has been used for a variety of applications, including protein design and engineering (reviewed in Yan *et al.*, 2014), as well as our own studies, unraveling the role played by key residues in FVIII and FIX deficiencies (Lopes *et al.*, 2021a, b, 2022). Here, we called this framework AT-RIN.

We used this network to study the properties of the different types of thrombosis of more than 130 patients displaying single-point mutations. We found that mutations causing Type I and Type II deficiencies (as well as its sub-types) display notable characteristics that reflect the location of where these mutations occur in the protein. Moreover, we built a customized machine learning framework to predict the risk of thrombosis in patients harboring non-synonymous mutations in the AT protein (we named it AT-Class), and once the risk has been determined, the AT-Class predicted the most likely type of AT deficiency. Importantly, we made the AT-Class framework available as an open source project, anticipating that the scientific community will use it and extend it to study other diseases.

Together, these findings demonstrate the feasibility of using powerful algorithms trained on the knowledge accumulated over several decades of research, to understand the inner workings of a protein that is the master regulator of blood coagulation.

## 2 Results

### 2.1 The AT protein network

**Fig. 1.**
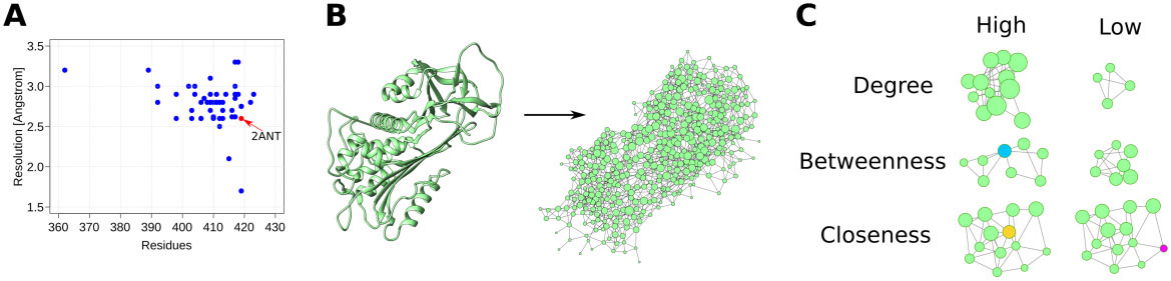
AT structures and the AT-RIN. (**A**) Each dot represents an AT structure obtained from the PDB. In red is the 2ANT structure, with a fair balance between the number of residues determined (419) and the structure resolution (2.6 Å). (**B**) To create the AT-RIN, we represented each residue as a node, and connected two nodes by an edge if the atoms of the amino acids were in close proximity to each other (∼5 Å), and took part in covalent or non-covalent interactions, as well as hydrogen-bonds (Section 4). (**C**) While nodes with low degree are connected to only a few others, nodes with high degree are taking part in interactions with multiple residues. A node displaying a high-betweenness value (blue) is central in the sense that it serves as a bridge between nodes that do not have direct connections between them; on the other hand, groups of residues that are connected to each other display low betweenness values. Finally, a node with a high closeness value (yellow) is able to reach every other node in the network with only a few steps, contrary to a node with low closeness value (pink), that is relatively distant from all other nodes

The AT protein does not exist in a single stable conformation, instead, it exists as an ensemble of conformations, that change depending on whether it is found as a monomer or bound to heparin or to one of its target factors (FIXa, FX and thrombin) (Huntington, 2011; Rezaie and Giri, 2020). To build a RIN of the AT to study the relation between single-point mutations and the occurrence of thrombosis, we first had to select the 3D structure with the best resolution and the most appropriate conformation. For this purpose, we investigated the 3D structures of 27 AT models deposited in the Protein Data Bank (PDB) and found that these models displayed a broad variety of resolutions and portions of missing residues (Fig. 1A and Supplementary Table S1). Based on these observations, we selected the model 2ANT as the basis for the construction of the AT-RIN. This model has only 12 missing residues and was determined at 2.6 Å (Skinner *et al.*, 1997). In all, these observations reflect the difficulty in determining the precise position of residues located in regions of high flexibility, and while future studies may benefit from AT structures at different conformations and displaying higher resolutions, at present, the 2ANT model is the most comprehensive model to serve as the foundation of the AT-RIN.

We used all 419 residues of the 2ANT model as nodes and connected two nodes if their main- or side-chains were located within ∼5 Å from each other (Fig. 1B and Supplementary Table S2). The resulting network had 419 nodes, more than 1500 edges and formed an undirected weighted graph, where the weights of the edges represent the strength and confidence of the interaction between two residues (Section 4). In this format, a complex structure became suitable as input for network analysis algorithms.

We wanted to understand the connectivity characteristics of the whole AT-RIN as well as identify the most connected and central residues of the whole network. For this purpose, we determined the connectivity characteristics of all residues of the AT-RIN using three different centrality measures that are commonly used in network analysis (Supplementary Table S3). The degree is simply the number of neighbors that a node has, the betweenness measures how many shortest paths rely on a given node when connecting all residues in the network to each other and starting at a given node, the closeness quantifies the number of steps necessary to reach every other node in the network (Fig. 1C). Together, these centrality measures quantify the importance of every residue of the AT protein in a local context (degree and betweenness) as well as from a global perspective (closeness), reflecting potential allosteric effects that propagate through the structure and result in conformation changes at distal sites (Censoni *et al.*, 2017; Guo and Zhou, 2016).

**Fig. 2.**
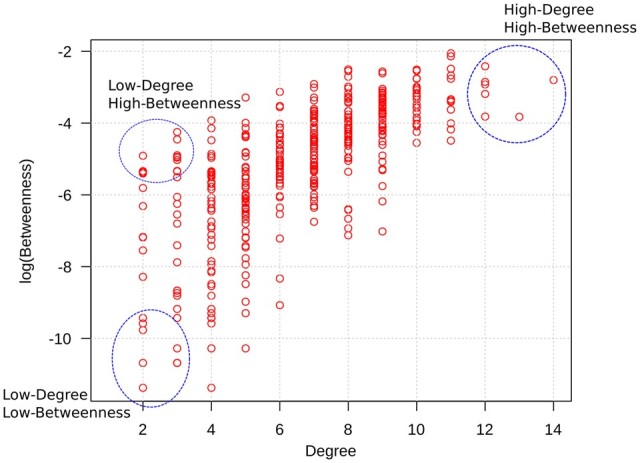
Relationship between the degree and the betweenness of residues. Each dot represents a residue of the AT protein and the centrality measures were derived from the AT-RIN. Similar to the residue networks derived from other proteins [13, 15], the AT RIN also displays residues that are highly central in the network (HDHB), serve as bridges between the different parts of the molecule (LDHB) or are peripheral nodes interacting with a few other residues and located at the surface of the protein (LDHB)

We observed that the centrality characteristics of the AT residues are similar to those of other proteins (Lopes *et al.*, 2021b, 2022), with a few residues interacting with several others, and numerous residues taking part in only a few interactions. In particular, when we consider the degree and the betweenness together, we immediately identify an emerging pattern in the AT structure, namely, the existence of residues with high-degree and high-betweenness (HDHB), residues with low-degree and high-betweenness (LDHB) and finally, residues displaying low-degree and low-betweenness (LDLB) (Fig. 2). The HDHB residues are usually located at the core of the AT protein, are hydrophobic and highly conserved. The LDHB residues are located at intermediate positions between the core of AT and its surface, and despite its low degree values, these residues serve as ‘bridges’ to maintain the connection between groups of residues that would not be connected otherwise. Finally, the LDLB residues found at the surface are polar and less conserved than the other residues. Together, these observations indicate that the centrality measures derived from the AT-RIN can quantify the importance of all of its residues; importantly, these measures agree with and reflect the properties of other well-known structural and evolutionary characteristics.

**Fig. 3.**
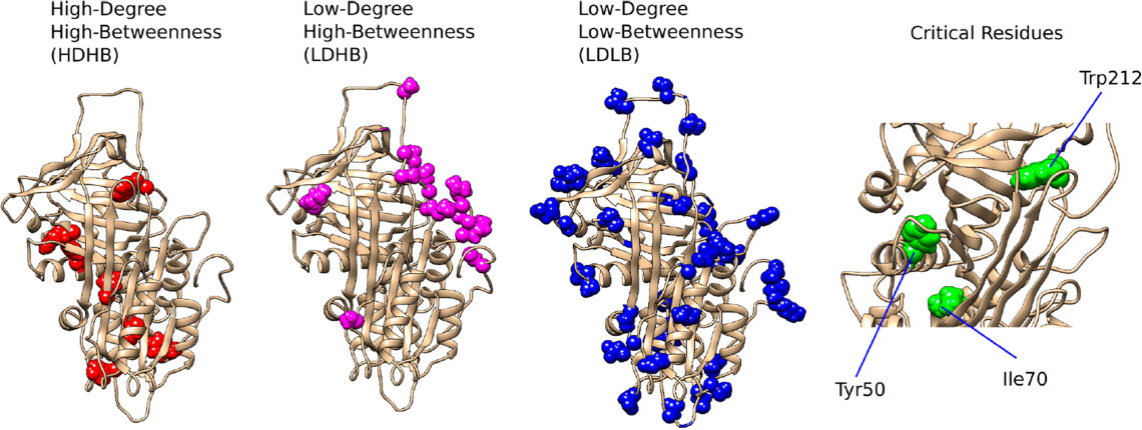
Mapping of residues with different network properties. Residues with HDHB are mainly located at the hydrophobic core of the structure and hold it in place via multiple attractive and repulsive forces to other numerous residues. Low-degree, high-betweenness residues are those that serve as bridges between groups of residues that would not be connected otherwise. On the other hand, residues with LDLB values are usually those at the surface of the protein and that do not have atomic connections to many others. Finally, the critical residues are those that have many neighbors, serve as bridges to several groups of residues and are located within a few steps from every other residue in the network (high-closeness values)

Finally, we used the degree, betweenness and closeness values in conjunction to identify the most critical residues of the AT protein. We used the Pareto front to identify the residues that had the highest values in at least one of the three measures, and found that three residues fell under these criteria (Fig. 3). Similar to the HDHB residues, the critical residues of AT are located at the structure core, are highly conserved and its main- and side-chains are located within less than 5 Å of more than 30 other residues. Similar to the hub stations of a metropolitan train network, these critical residues play a pivotal role in maintaining the stability of the network, and any perturbations (i.e., mutations) inevitably lead to the disruption of the whole network (Han, 2008; Yan *et al.*, 2014).

### 2.2 Mapping of mutation data

**Fig. 4.**
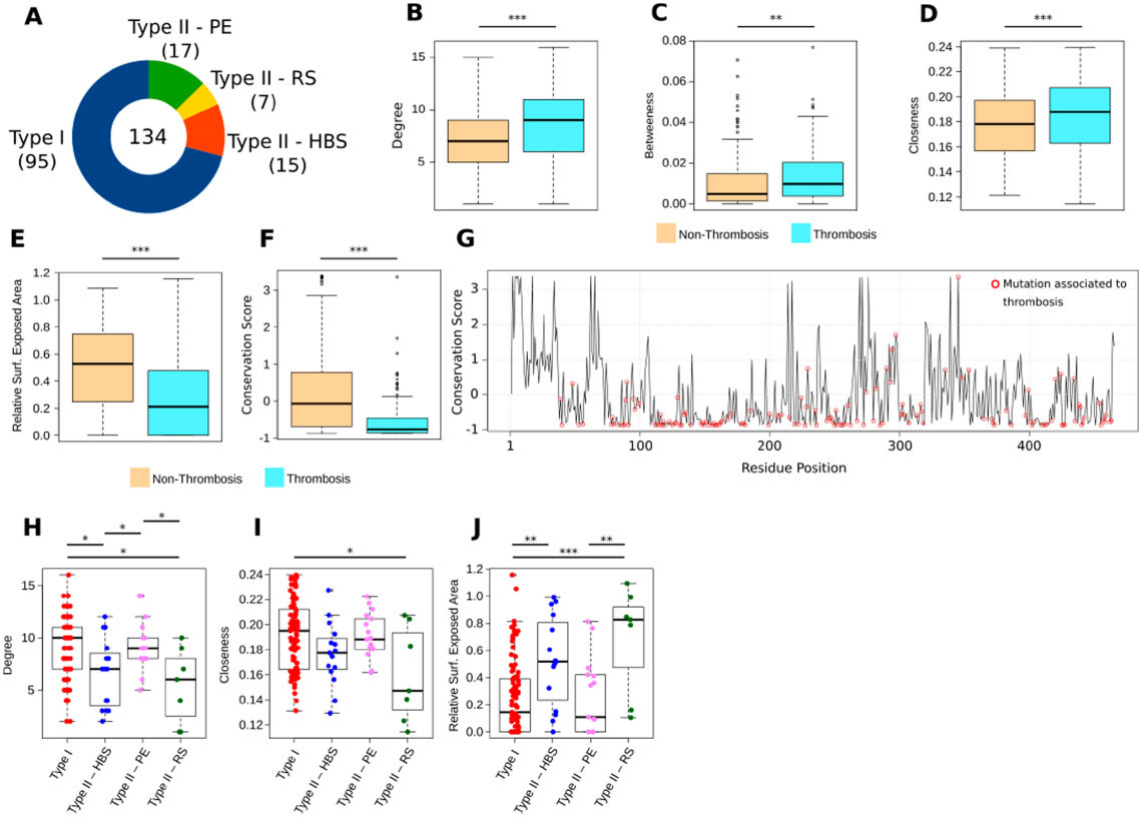
Characteristics of thrombosis-related mutations. (**A**) The number of cases and thrombosis types in our dataset (see Section 4). (**B**–**G**) Comparison of the characteristics from the residues where substitutions lead to thrombosis versus the residues without reports in the medical literature relating them to the occurrence of thrombosis (*n* = 134 versus *n* = 300). These characteristics were derived from the 3D structure of the human AT (PDB code 2ANT) and from network centrality characteristics from the AT-RIN (see Section 2). Additionally, the conservation score was derived from a multiple sequence alignment of more than 400 species (Section 4). (**H**–**J**) Characteristics that can distinguish between the multiple thrombosis subtypes. In all cases, the boxplots depict the median (center line), the first and third quartiles (lower and upper bounds) and 1.5 times the inter-quartile range (lower and upper whiskers). Each dot in the plot is a residue mutation. Unpaired, two-sided Wilcoxon test (***p-value < 0.001; **p-value < 0.01; *p-value < 0.05)

After obtaining centrality measures from the AT-RIN, we wondered what are the characteristics of the residues that lead to thrombosis if substituted? For this purpose, we collected the mutation profile of 134 patients from different countries as well as their thrombosis type (Type I, Type II HBS, Type II RS and Type II PE; Fig. 4A and Supplementary Table S4). The data were obtained from the professional version of the Human Gene Mutation Database (HGMD), an initiative where the biocurators manually collect, verify and organize the mutations reported in the biomedical literature; these mutations relate several types of genetic modifications to disease phenotypes (https://www.hgmd.cf.ac.uk). With the data existent at present, we were only able to consider single-point non-synonymous mutations leading to thrombosis (the number of documented cases of large genetic perturbations to SERPINC1 and the resolution of current protein structure modeling methods is not sufficient to create an insertion/deletion dataset of appropriate size).

We prepared slightly modified versions of the AT-RIN (Section 4), and upon mapping into these networks, the residues whose mutations lead to Type I thrombosis, we observed that they are mainly located at the core of AT, are central interaction points of several residues and are highly conserved (Fig. 4B–G). These observations largely explain why perturbations of these residues lead to detrimental effects in the protein stability and conformation, and cause the low expression that is characteristic of Type I deficiency (Bayston and Lane, 1997; Lichtman *et al.*, 2021; Luxembourg *et al.*, 2011).

We observed that residues whose mutations lead to Type II deficiency were less conserved, mainly located at the surface of AT and were connected to a lower number of residues than their Type I counterparts. This is unsurprising, given that Type II deficiency is characterized by mutations that hamper the heparin and target binding functions of AT—mainly performed by the interactive sites at the surface of proteins. However, we found that several of those residues were not directly located at the surface of A, but at the layer ‘below’ the heparin and RSs of AT (Fig. 4C), and without mutations, these residues are likely to hold the binding site residues at its correct orientation through attractive and repulsive forces (del Sol *et al.*, 2006; Reichmann *et al.*, 2005).

Next, given that the AT-RIN was created based on the conformation of AT in its native state, we wondered if these observations would be reflected in a RIN created using its latent or hyperstable conformation. For this purpose, we followed the same steps to create a native RIN, and generated a RIN of the chain I of the 2ANT structure. We verified that although the centrality measures of the networks in its latent or native state resemble each other closely, the latent network characteristics are not suitable to distinguish between the different types of deficiency, suggesting that AT’s native fold is better suited to anticipate the effect of point-mutations (Supplementary Fig. S1).

Finally, we observed that while we could readily distinguish the characteristics of residues whose mutations lead to Type I and Type II HBS or RS deficiency, Type I and Type II PE residues could not be distinguished by any measures. Even though we had only 17 cases of Type II PE in our study population, this suggests that Type I PE residues have characteristics that resemble Type I deficiency more closely than the residues whose mutations lead to Type II deficiency (Fig. 4H–J).

Taken together, these results demonstrate the suitability of using an *in silico* representation of the AT protein to study the properties of residues that lead to different types of AT deficiency. Moreover, it is reassuring that the same conclusions were derived from measures that quantify key aspects of the AT protein from different perspectives, including its structural and evolutionary aspects.

### 2.3 Predicting the risk of thrombosis

After understanding the characteristics of the residues whose substitutions lead to different types of thrombosis, we aimed to use all centrality, structural and evolutionary measures in conjunction to predict the risk of thrombosis in patients harboring non-synonymous mutations. For this purpose, we established a machine learning framework that uses the characteristics of the residues as input data (the so-called ‘training set’) and outputs the risk of thrombosis (on a scale from 0 to 1). We named this framework AT-Class.

ML algorithms are known to require large amounts of input data to derive meaningful patterns and generalize to unseen cases; however, our dataset had notorious characteristics that are problematic for ML algorithms: first, a small number of reported thrombosis cases and second, unbalanced classes (∼130 mutations related to thrombosis versus ∼300 residues without reports in the medical literature). To deal with this challenge, we developed a training and testing procedure that uses only part of the instances and a single feature at a time; we repeated these steps hundreds of times and combined all predictions at the end (Section 4). This robust training scheme resembles an ensemble of classifiers in the sense that it uses different portions of the dataset at each training step. To evaluate our results, we used the area under the ROC curve (AUC), that quantifies the balance between true- and false-positive predictions, and the F1-score, which is the harmonic mean of the precision and the recall of the classification (i.e. it measures how sensitive the classifiers are to assign thrombosis and non-thrombosis cases to their specific classes). In both cases, 0.5 is the baseline for random predictions and 1.0 represents perfect prediction of all instances.

**Fig. 5.**
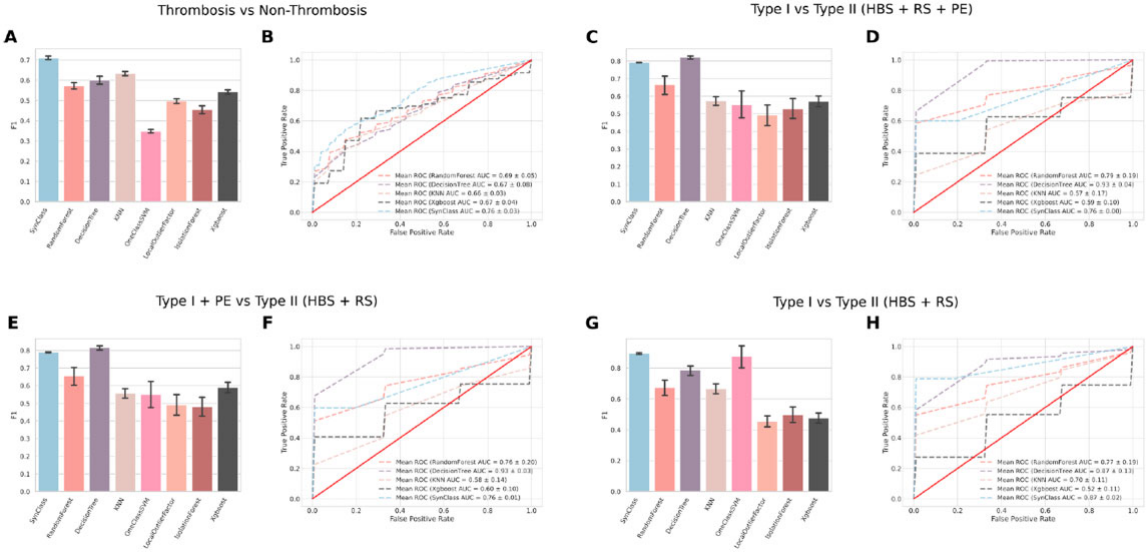
The AT-Class machine learning framework. (**A**) The F1 measures of different ML classifiers predicting the occurrence or non-occurrence of thrombosis. The F1 is the harmonic mean between the precision and the recall measures; higher values indicate better performance. (**B**) The AUC of the classifiers; values close to 1.0 indicate that the classifiers could correctly detect true-positives while rejecting false-positives. (**C** and **D**) The same classification measures as in the previous panels, but predicting the occurrence of Type I versus Type II deficiency. Here, Type II cases encompass HBS, RS and PE subtypes. (**E** and **F**) The same classification measures as in the previous panels, but predicting the occurrence of Type I versus Type II deficiencies. Here, Type I and Type II PE cases were combined and Type II cases encompass only HBS and RS. (**G** and **H**) The same classification measures as in the previous panels, but predicting the occurrence of Type I versus Type II deficiencies, removing the Type II PE cases from the dataset. The bars indicate the mean of 50 executions of the algorithm, and the error bars depict the standard deviation of those measurements. SVM: support vector machine.

In our case, using the robust training and testing procedure and different classifiers, we were able to predict the incidence of thrombosis with AUC and F1-score of 0.70 and 0.76, respectively (Fig. 5A and B). Compared with other classification strategies, this adaptive classification yielded remarkably better results, and demonstrated that structural, evolutionary and centralities measures can assess the risk of thrombosis in patients with single non-synonymous point mutations.

Next, we wondered if we could predict the most likely type of thrombosis using the same features from the previous classification. We divided our dataset into two classes, namely, Type I deficiency versus Type II deficiency, where the Type II instances comprised the RS, HBS and PE cases. However, given our observation that the characteristics of the residues whose mutations lead to Type I and Type II PE were similar (Fig. 4C), we created additional datasets to assess the ML classification performance and to obtain additional insights about the disease biology. Once more, using our robust training and testing regimen, the algorithms obtained a F1-score of 0.8 and AUC of 0.93 when predicting the occurrence of Type I or Type II (HBS + RS + PE) cases (Fig. 5C and D). These values were similar if we considered Type I and Type II PE cases together versus Type II (HBS + RS) cases (Fig. 5E and F) and if we removed altogether the PE cases from the classification [F1: 0.86 and AUC: 0.87 (Fig. 5G and H)]. This reinforces that the molecular characteristics of the Type I and Type II PE deficiency cases are similar and most likely indistinguishable from each other. As reported previously, these mutations lead AT to a premature transition to its latent state, which impair its heparin and protease binding properties (de la Morena-Barrio *et al.*, 2017).

In terms of performance, although our algorithms had better results than other methods (Adzhubei *et al.*, 2013; Flanagan *et al.*, 2010) at anticipating the effects of single-point mutations (Supplementary Figs S2 and S3), at present it is not possible to anticipate which of our classifier algorithm will perform better in which dataset (i.e. thrombosis versus non-thrombosis and Type I versus Type II deficiencies); therefore, we compared the performance of the most established algorithms in the field. A tentative explanation for the difference in performance might be the difference in the number of instances in the datasets, and in the non-linear relationships created by when considering all attributes in conjunction (Bishop and Nasrabadi, 2006; Goodfellow *et al.*, 2016).

In summary, we found that it is feasible to use ML algorithms to predict the risk of thrombosis, and given the position of a single-point mutation in the AT protein, we can anticipate the most likely type of deficiency that will occur. While the number of cases reported in the literature prevents us from performing additional analyses that are clinically relevant (e.g., Type II HBS versus all others), we regard as encouraging the fact that the AT-Class was able to achieve a relatively high accuracy even with the small number of cases available at present.

## 3 Discussion

In this study, we introduce a complete mapping of the AT protein that effectively quantifies the importance of its residue. We found that the position of the residues within the residue network is largely associated with its evolutionary conservation, as well as its location at the core or at the surface of the protein structure. The impact of this novel representation is 2-fold; first, it can be used as input to a robust machine learning framework to assess the risk of thrombosis in patients and second, it serves as a roadmap to support the development of recombinant AT proteins, indicating in precise quantitative terms which residues are safe to substitute.

While it is clear that large deletions and substitutions to AT impair its function and prevent it from even leaving the endoplasmic reticulum (Bayston and Lane, 1997; Lichtman *et al.*, 2021; Luxembourg *et al.*, 2011), the effect of single-point mutations is harder to anticipate—especially considering only the protein sequence. Therefore, by using the AT protein structure, we examined the spatial characteristics of each residue, and by transforming the structure into a network, we quantified the connectivity of these residues. While it is common knowledge in the structural biology field that ‘residues at the protein core are hydrophobic and disrupt the structure if mutated’, this is largely a qualitative statement; here, we quantified the importance of every AT residue from multiple perspectives, and as our results demonstrate, these values are largely associated to the type of thrombosis that is more likely to occur upon mutation (Fig. 4).

While explaining the reason underlying Type I and Type II deficiency of known cases is important, we also wanted to predict the risk of thrombosis in patients harboring previously unseen mutations. For this purpose, we used all measures in conjunction as input for the AT-Class framework (Fig. 5). Interestingly, even considering the small dataset available at present, the AT-Class framework was able to achieve high classification performance, indicating that the molecular features that characterize Type I and Type II deficiency are highly specific and can be distinguished clearly (Fig. 5).

Overall, this study opens exciting research avenues for coagulation research. First, we only considered AT in one of its conformations, but as we know, it exists in different conformations and in complex to its targets. Therefore, creating residue networks of these states might explain even further the biology of AT and its role as a critical serine protease inhibitor. Moreover, from the clinical and therapy development point of view, it would be very useful to anticipate the effect of benign or gain-of-function mutations in AT, although we understand that existing algorithms are better suited to predict harmful effects of residue substitutions (Broom *et al.*, 2020). Second, as the number of human genomes sequenced is increasing dramatically, it will be interesting to assess the risk of thrombosis in the general population and to monitor specific cases even before they manifest any symptoms. Third, future versions of the AT-Class should consider the effect of non-synonymous as well as substitutions that occur at introns, as these mutations affect the overall RNA structure and splicing (Vaz-Drago *et al.*, 2017), and importantly, are associated to AT deficiency (Mulder *et al.*, 2017; Toderici *et al.*, 2016). Finally, the occurrence of thrombosis has a relation to other factors, and there are deficiencies with high and low risk of thrombosis. Therefore, measuring the levels of known blood markers (e.g., d-dimer, activated partial thromboplastin time, platelet counts, FVIII and FIX activity), as well as including family history of thrombosis cases, will certainly leverage the predictive power of AT-Class.

In summary, our results demonstrate how a framework combining multiple levels of molecular, clinical and computational techniques enhances diagnosis and can aid the design of novel recombinant proteins. Importantly, by making all source code available to the community, we expect that the AT-Class will be promptly retrained as novel information becomes available and that it will be repurposed to study other diseases.

## 4 Materials and methods

### 4.1 Protein structure and network construction

We downloaded the 3D structure of AT (PDB: 2ANT; Skinner *et al.*, 1997) and preprocessed only chain I by renumbering its residues to start at 1. Next, we performed a side-chain readjustment (also known as ‘relax’) using Rosetta (Leaver-Fay *et al.*, 2011). We selected the model with the lowest free energy for further analyses.

We used RINerator version 0.5.1 (Doncheva *et al.*, 2011) to create the RINs. We created different versions of the AT-RIN, depending on the thrombotic phenotypes we compared. First, we normalized the score provided by RINerator to the interval [0, 1]. Next, we subtracted these values from 1 (namely, 1—normalized_score). This ensured that higher values represented stronger atomic interactions. Finally, we considered only interactions with values higher than 0.1. The complete networks are available in Supplementary Table S2, together with the definitions of the types of interactions used to build each network.

From these networks, we calculated the degree, the betweenness and the closeness values of each node using the R statistical package version 4.2.0 (www.r-project.org) and package iGraph version 1.2.5 (Csardi *et al.*, 2006).

### 4.2 Other relevant measures

We used Chimera version 1.14 (Pettersen *et al.*, 2004) to extract the solvent-excluded area (areaSES) and to calculate the relative surface exposure of all amino acids from the relaxed 2ANT structure (chain I). We divided the solvent-excluded area of the residue by the surface area of the same type of residue in a reference state; in our case, we used the reference values of the 20 standard amino acids in Gly-X-Gly tripeptides (Bendell *et al.*, 2014). Other measures calculated with Chimera were areaSAS, kdHydrophobicity, PSI and PHI, for each residue of the 2ANT structure.

We obtained the conservation score from the ConsurfDB webserver (Ben Chorin *et al.*, 2020), using the 2ANT structure and an alignment 417 sequences from AT in other species as input for the search query (Supplementary Table S4).

### 4.3 Mutation profile of patients

We collected the AT mutation profile of 134 patients from the professional version of the HGMD (www.hgmd.cf.ac.uk). We manually processed each record and inspected the associated publication describing the case reports; this was necessary to determine the thrombotic phenotype (e.g. Type I and Type II HBS). We selected only the cases caused by single-point mutations and considered only one report per mutation (Supplementary Table S4). The reason why we did not consider multiple reports per mutation was not to inflate the training and test sets; namely, each mutation became an instance for those datasets, and because some mutations appear more frequently than others, they would bias the classification toward those instances.

### 4.4 Machine learning methods

In this work, the learning is focused on identifying patterns in patients’ data and predicting the development of thrombosis and its particular types. To reach this goal, we established a ML pipeline that follows three well-defined steps. First, the patients’ data, formally referred to as input space X, were preprocessed to remove missing values.

In the second step, we designed our experiments by randomly splitting into training (80%) and test (20%) folds. To avoid drawing biased conclusions, this process was randomly repeated 50 times and the final results were discussed in terms of mean and standard deviation. During the training phase, the search for the optimal model configurations was induced by combining the stratified 10-fold cross-validation approach and the random search strategy, which is a hyper-parameter optimizer implemented to find models by effectively searching a larger configuration space that even includes less promising possibilities (Bergstra and Bengio, 2012).

In our study, we considered supervised and unsupervised ML methods. In general, supervised methods are seen as functions (*f*) responsible for mapping an input space to labels Y as the relation f:X→Y. The unsupervised methods, in turn, work without considering Y, that is, only the input space is analyzed to find relevant partitions. Among the supervised methods, we used the K-nearest neighbors (KNN) (Bishop and Nasrabadi, 2006), decision trees (DT) (Breiman *et al.*, 1984), XGBoost (Chen and Guestrin, 2016) and random forest (RF) (Breiman, 2001). In relation to the unsupervised methods, we considered, isolation forest (Liu *et al.*, 2008), local outlier factor (LOF) (Breunig *et al.*, 2000) and one class support vector machine (Schölkopf *et al.*, 2001).

The KNN model was adjusted by varying the number of neighbors from 1 to 25 and the Minkowski distance from 1 to 4. Moreover, we also considered the distance-weighted nearest neighbors (DWNN) (Bishop and Nasrabadi, 2006) by weighting the class votes according to the neighbor distances. The DT model was estimated by varying the minimum number of observations in a terminal node (leaf) and the maximum depth within the interval [1, 25]. We also evaluated two different functions to measure the node importance: Gini and Entropy. Next, we evaluated two ensemble methods based on DT. XGBoost was estimated by varying the maximum depth of a tree inside the interval [1, 25], the learning rate by scaling the contribution of each tree as η∈[0.05,0.30], the minimum sum of instance weight in a child in the interval [1, 7], the minimum loss reduction to split a leaf node between [0.0, 0.4] and the subsample ratio of columns inside the interval [0.3, 0.7]. The RF ensemble was trained by using the maximum depth within the interval [1, 25] and the number of trees between 1 and 25.

The Isolation Forest was trained by varying the number of base models from 10 to 100, the proportion of outliers in the dataset between 0.1 and 0.5, the number of patients’ features from 2 to the maximum and the presence/absence of bootstrap (training data created sampled with replacement). LOF was estimated by considering the number of neighbors from 5 to 45, the proportion of outliers in the dataset between 0.35 and 0.5 and the Minkowski distance from 1 to 5. All hyper-parameter values were empirically defined by analyzing every model according to the extrema points limiting the general performance, that is, the minimum and maximum values were defined when the performance significantly dropped.

As discussed in Section 2, the best overall performance was obtained using DT. In this sense, we decided to use an ensemble that combines different DT estimated on every individual attribute from the dataset (this ensemble is referred to as SynClass). From such trees, we combined the individual predictions by considering weights provided by applying the Wilcoxon test comparing the values from each attribute and the two different classes. The weights of the classifications of each feature were combined along with the outcomes yielded from every tree to decide the final class.

In our ML pipeline, the last step was achieved by assessing the best models on the test folds. To mitigate the impact of imbalanced data, besides using stratified cross-validation, we created test folds with the same number of patients for each expected label Y. The validation methods considered in our experiments were the F1-score and the AUC. The F1-score was used not only to assess the model performances on the test folds but also to induce the estimated models during the training phase.

In our experiments, all codes were written in Python version 3.9.12 and scikit-learn version 1.0.2 to build up the ML models, training tasks and validation methods.

For comparison, we used our mutation datasets as input to Polyphen-2 (Adzhubei *et al.*, 2013) and to SIFT (Flanagan *et al.*, 2010).

## Supplementary Material

vbac098_Supplementary_Data

## Data Availability

The source code and datasets used in this study are available at https://github.com/madlopes/AT-Class.
