# Supplementary figures and images for: Computational analyses reveal fundamental properties of the AT structure related to thrombosis
Source: Bioinform Adv. 2022 Dec 23;3(1):vbac098. doi: 10.1093/bioadv/vbac098 (PMC9838315; doi:10.1093/bioadv/vbac098)

SIFT Score

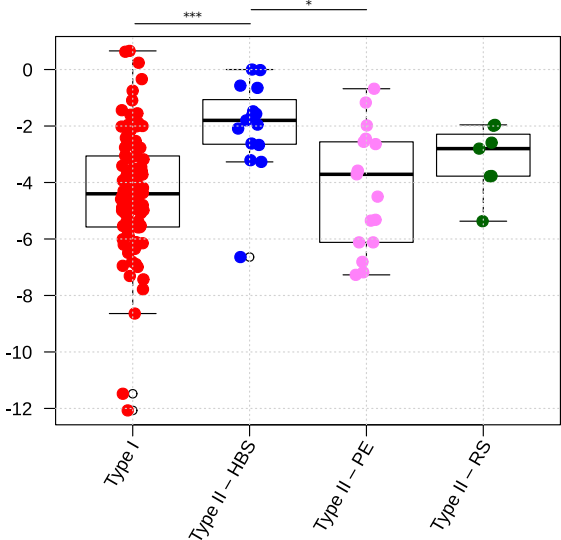

Supplement: vbac098_Supplementary_Data [file vbac098_supplementary_data.zip › Supplementary Figure 3 - SIFT.pdf]

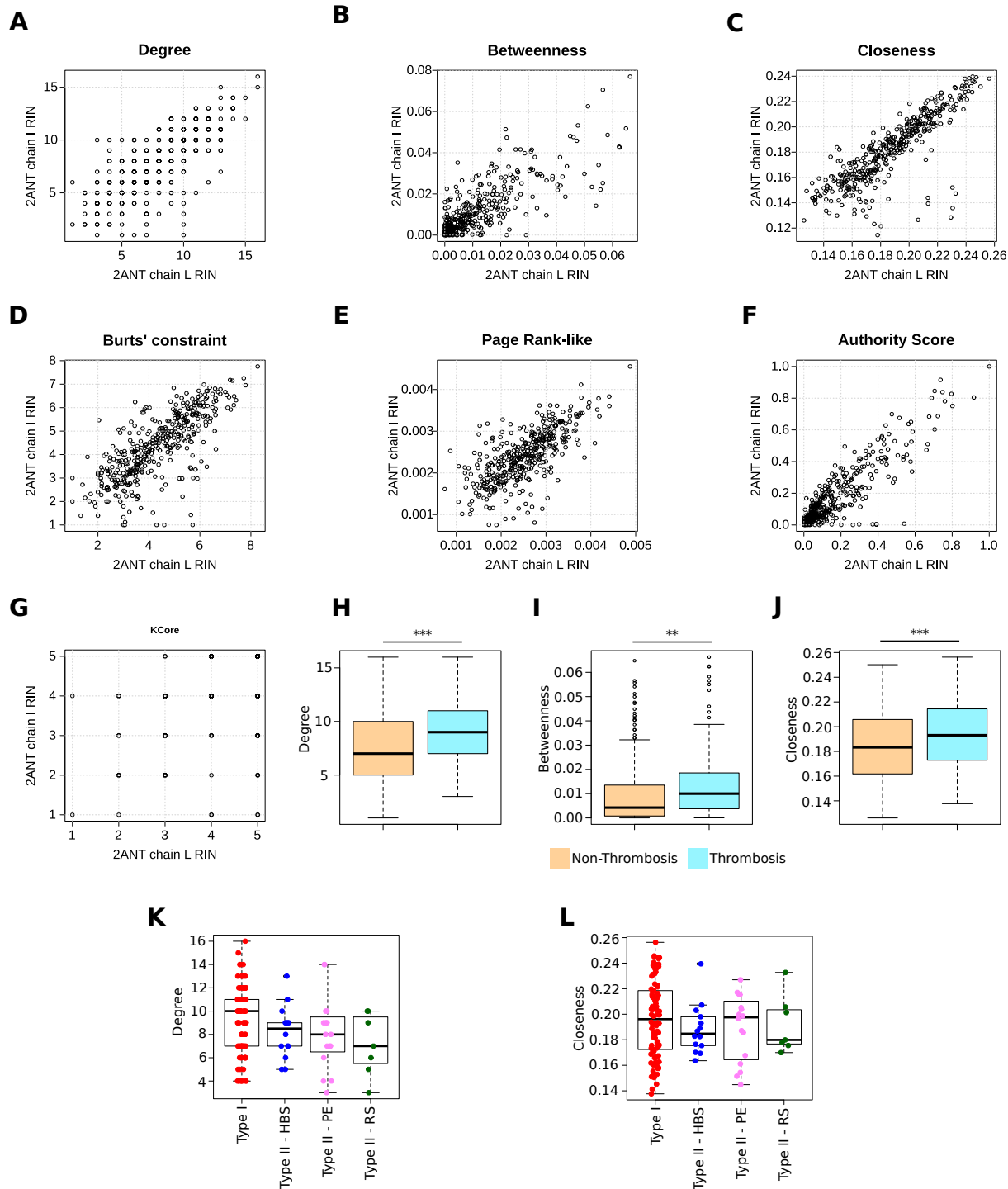

Supplement: vbac098_Supplementary_Data [file vbac098_supplementary_data.zip › Supplementary Figure 1 - Boxplots chain comparisons.pdf]

**A**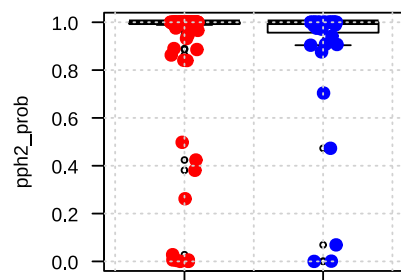**B**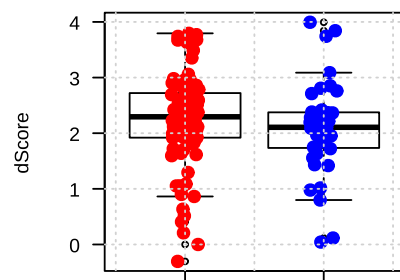**C**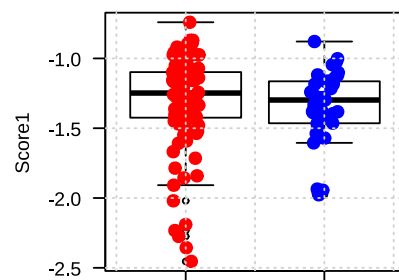**D**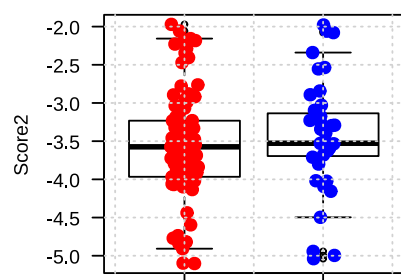**E**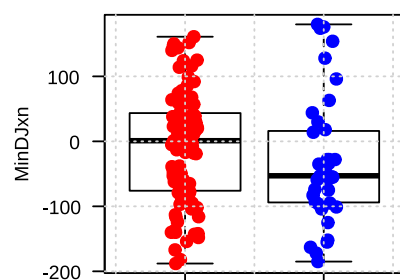**F**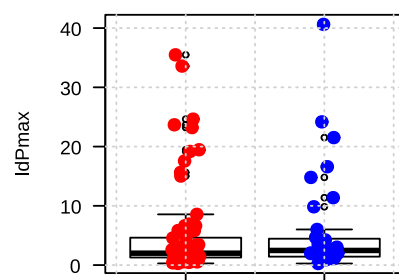**G**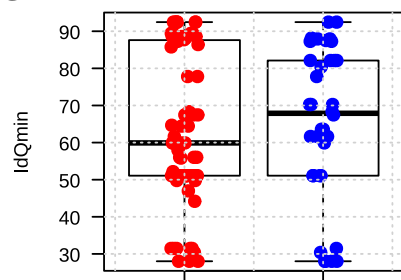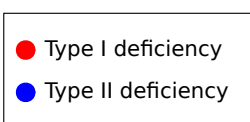

Supplement: vbac098_Supplementary_Data [file vbac098_supplementary_data.zip › Supplementary Figure 2 - Polyphen2.pdf]
